# Complete genome sequence of *Clostridium butyricum* DKU-11, isolated from healthy infant feces

**DOI:** 10.1128/mra.00037-24

**Published:** 2024-04-23

**Authors:** SangJoon Mo

**Affiliations:** 1 Department of Microbiology, Dankook University, Chungnam, Cheonan, South Korea; 2 Center for Bio-Medical Engineering Core Facility, Dankook University, Chungnam, Cheonan, South Korea; Rochester Institute of Technology, Rochester, New York, USA

**Keywords:** anaerobic bacteria, probiotics, gut microbiota, endospores

## Abstract

*Clostridium butyricum* DKU-11, a bacterium has been isolated from the stools of breastfed infants near Cheonan-Asan city, Republic of Korea, and has a genome sequence of 4,630,814 bp. The GC content is 28.7% and a total of 4,137 coding sequences in two contigs.

## ANNOUNCEMENT

Stabilization of the human gut microbiota is an indispensable topic for discussing not only intestinal diseases but also the occurrence of various diseases (1). Independent metabolites and enzymes of obligate anaerobic *Clostridium butyricum* have been reported to regulate probiotic properties, intestinal immune function, and gastrointestinal barrier function, and recently it has been involved in the generation of neurotransmitters that affect mood and cognitive function (2, 3). In this study, we describe the entire genome sequence of *C. butyricum* DKU-11 isolated from infant stools. Stools were collected from a gynecologic hospital and postpartum care center in Cheonan-Asan with Dankook University Hospital Clinical Trial Committee review (IRB File NO. 2013–01-007-001).

To isolate *C. butyricum* from infant stool, the collected stool samples were placed in 5 mL of Reinforced Clostridial Medium (RCM, BD Difco, USA) liquid medium with the top of the medium covered with liquid paraffin and incubated for 3 days at 30°C in an anaerobic chamber. Afterward, the final *C. butyricum* was selected by analyzing the 16S rRNA base sequence of the isolated strain. Butyric acid was analyzed using high-performance liquid chromatography (HPLC). The mobile phase was a mixture of 10 mM phosphate buffer (pH 3.0) and methanol at a ratio of 98:2, and HPLC was operated at a flow rate of 1.0 ml/min and a UV wavelength of 220 nm. The concentration of butyric acid was measured using a calibration curve. Among the isolated strains, DKU-11 was selected as the strain with the highest butyric acid content. The isolated DKU-11 strain was cultivated under anaerobic conditions as described in a previous study (4), and genomic DNA was obtained using a DNA isolation kit (Maxwell Prokaryote/Eukaryote SEV DNA Purification Kit, Promega, USA) according to the manufacturer’s directions. The whole genome of DKU-11 was sequenced using the Pacific Biosciences (Menlo Park, USA) Sequel platform and Illumina (San Diego, USA) HiSeq X Ten platform at Macrogen Inc. (Republic of Korea). For PacBio Sequel II HiFi sequencing (Pacific Biosciences, USA), the SMRTbell template was used, following the standard PacBio 7–12kb library preparation procedure (5). For Illumina analysis, sequencing libraries were prepared by ligation of randomly fragmented DNA samples into 5′ and 3′ adapters according to the manufacturer’s TruSeq Nano DNA High Throughput Library Prep Kit (Illumina, USA) instructions which were then used for curation of raw data to perform further analysis. A complete genome was generated with a *de novo* hybrid strategy. *De novo* assembly of the PacBio raw reads was first performed using the Microbial Genome Assembly application in SMRTlink 11.0.0.146107 [based on Hierarchical Genome Assembly Process (HGAP) version 4 (6)] and after assembly, polishing was performed using Racon v1.5.0 (7). The Illumina raw reads were used to correct errors by DNA bases accurately, fixing mismatches, and assembly rearrangements using Pilon v1.21 (https://github.com/broadinstitute/pilon/wiki) (8). The NCBI Prokaryotic Genome Annotation Pipeline (PGAP) v6.1 was used for annotation (9).

Annotation results for the DKU-11 genome through *de novo* assembly are given in Table 1. The entire genomes of the DKU-11 consist of two contigs amounting to 4,852,175 bp. They contain putative genes associated with predation (e.g., various carbohydrate metabolic process enzymes, sporulation-specific proteins). Further genome analysis will help elucidate the biochemistry, physiology, probiotic mechanisms, and ecological functions of anaerobic organic-acid-producing bacteria.

**TABLE 1:** Sequencing, assembly, and annotation results for the whole genome sequenced *C. butyricum* DKU-11 strain

Category	Feature	*C*. *butyricum* DKU-11
HiFi reads	No. of reads	83,754
	Total bases (bp)	766,124,287
	N50 (bp)	10,438
Filtered Illumina reads	No. of reads	8,119,598
	Total bases (bp)	1,225,066,271
	Q30 (%)	98.47
Assembly	No. of contigs	2
Contig 1	Length (bp)	3,861,517
Contig 2	Length (bp)	769,297
Total	Genome size (bp)	4,630,814
	N50 (bp)	3,861,510
	GC content (%)	28.7
	Mean depth (×)	165.3
	No. of CDSs	4,137
	Coding ratio (%)	96
	No. of rRNAs	33
	No. of tRNAs	88
Accession data	Biosample accession no.	SAMN35992232
	BioProject accession no.	PRJNA987805
	GenBank accession no.	CP128832.1-CP128833.1
	SRA accession no.	SRR27400125-SRR27400126

## Data Availability

The complete whole-genome sequence of *Clostridium butyricum* strain DKU-11 has been committed at DDBJ/ENA/GenBank under accession no. CP128832.1 and CP128833.1. In addition, accession numbers that can be used in GenBank are summarized in Table 1. This strain has been deposited in the Korean Collection for Type Cultures (KCTC) with accession number KCTC19096P.
